# Supplementary material for: Neutrophil infiltration regulates clock-gene expression to organize daily hepatic metabolism
Source: eLife. 2020 Dec 8;9:e59258. doi: 10.7554/eLife.59258 (PMC7723411; doi:10.7554/eLife.59258)
Supplement: Transparent reporting form [file elife-59258-transrepform.docx]

***eLife’s* transparent reporting form**

We encourage authors to provide detailed information *within their submission* to facilitate the interpretation and replication of experiments. Authors can upload supporting documentation to indicate the use of appropriate reporting guidelines for health-related research (see [EQUATOR Network](http://www.equator-network.org/%20)), life science research (see the [BioSharing Information Resource](https://biosharing.org/)), or the [ARRIVE guidelines](http://www.plosbiology.org/article/info:doi/10.1371/journal.pbio.1000412) for reporting work involving animal research. Where applicable, authors should refer to any relevant reporting standards documents in this form.

If you have any questions, please consult our Journal Policies and/or contact us: [editorial@elifesciences.org](mailto:editorial@elifesciences.org).

**Sample-size estimation**

- You should state whether an appropriate sample size was computed when the study was being designed
- You should state the statistical method of sample size computation and any required assumptions
- If no explicit power analysis was used, you should describe how you decided what sample (replicate) size (number) to use

Please outline where this information can be found within the submission (e.g., sections or figure legends), or explain why this information doesn’t apply to your submission:

To choose the size of the sample we have used the 3R rule to ensure statistical validity and significance with the chosen size.

CNIC has biostatisticians to help in designing our animal experiments. They use the most up-to-date statistical methods to ensure that the correct number of animals will be employed in each experiment. The number of animals in each group is determined by the statistical power that is required to detect significant biologically relevant differences. A meaningful difference in means can be detected using a t-test, assuming normality. We use 5-17 mice per group for at least 80% power for one- and two-sided testing.

The exact sample size and number of replicates are indicated in every figure legend. For animal research and in vitro experiments, data were obtained from at least 2-3 independent experiments with high consistency between samples.

**Replicates**

- You should report how often each experiment was performed
- You should include a definition of biological versus technical replication
- The data obtained should be provided and sufficient information should be provided to indicate the number of independent biological and/or technical replicates
- If you encountered any outliers, you should describe how these were handled
- Criteria for exclusion/inclusion of data should be clearly stated
- High-throughput sequence data should be uploaded before submission, with a private link for reviewers provided (these are available from both GEO and ArrayExpress)

Please outline where this information can be found within the submission (e.g., sections or figure legends), or explain why this information doesn’t apply to your submission:

At least each experiment was performed 2-3 times to validate its reproducibility. The number of biological replicates is indicated in every figure legend. Criteria for exclusion/inclusion in human samples are detailed in the Material and Methods section. Animals that presented disease or had been bitten because fight in the cage were excluded.

**Statistical reporting**

- Statistical analysis methods should be described and justified
- Raw data should be presented in figures whenever informative to do so (typically when N per group is less than 10)
- For each experiment, you should identify the statistical tests used, exact values of N, definitions of center, methods of multiple test correction, and dispersion and precision measures (e.g., mean, median, SD, SEM, confidence intervals; and, for the major substantive results, a measure of effect size (e.g., Pearson's r, Cohen's d)
- Report exact p-values wherever possible alongside the summary statistics and 95% confidence intervals. These should be reported for all key questions and not only when the p-value is less than 0.05.

Please outline where this information can be found within the submission (e.g., sections or figure legends), or explain why this information doesn’t apply to your submission:

Statistical methods and details related to statistical analysis are specified in Materials and Methods section of the main text. All numerical data are presented as mean ± SEM. In every figure legend the statistic test applied in each experiment and the p-value are indicated (95% confidence intervals).

For comparisons between two groups, the Student’s t-test was applied. For data with more than two data sets, we used one-way ANOVA coupled with Turkey’s multigroup test. When variances were unequal, Welch’s test or Kruskal-Wallis test coupled with Dunn’s multiple comparison test were applied, respectively. Multiple group comparisons in the rhythmicity of neutrophil infiltration were analyzed with two-way ANOVA followed by Fisher’s post hoc test. Linear relationships between variables were tested using Pearson’s correlation coefficient.

(For large datasets, or papers with a very large number of statistical tests, you may upload a single table file with tests, Ns, etc., with reference to sections in the manuscript.)

**Group allocation**

- Indicate how samples were allocated into experimental groups (in the case of clinical studies, please specify allocation to treatment method); if randomization was used, please also state if restricted randomization was applied
- Indicate if masking was used during group allocation, data collection and/or data analysis

Please outline where this information can be found within the submission (e.g., sections or figure legends), or explain why this information doesn’t apply to your submission:

For animal research, the cages were randomly allocated in our animal facility.

For the human cohort, participants were selected from patients who underwent laparoscopic cholecystectomy for gallstone disease.

Technicians were blinded analyzing samples and most of the studies were performed in such condition, except in some experiments (e.g. western blots) where samples were needed to be loaded correctly.

**Additional data files (“source data”)**

- We encourage you to upload relevant additional data files, such as numerical data that are represented as a graph in a figure, or as a summary table
- Where provided, these should be in the most useful format, and they can be uploaded as “Source data” files linked to a main figure or table
- Include model definition files including the full list of parameters used
- Include code used for data analysis (e.g., R, MatLab)
- Avoid stating that data files are “available upon request”

Please indicate the figures or tables for which source data files have been provided:

Source data is provided for the following figures:

Figure 1

Figure 2

Figure 3

Figure 4

Figure 5

Figure 1-figure supplement 1

Figure 1-figure supplement 2

Figure 2-figure supplement 1

Figure 2-figure supplement 2

Figure 3-figure supplement 1

Figure 4-figure supplement 1
